# Supplementary material for: Study protocol for the Innovative Support for Patients with SARS-COV-2 Infections Registry (INSPIRE): A longitudinal study of the medium and long-term sequelae of SARS-CoV-2 infection
Source: PLoS One. 2022 Mar 3;17(3):e0264260. doi: 10.1371/journal.pone.0264260 (PMC8893622; doi:10.1371/journal.pone.0264260)
Supplement: S1 File — (DOCX) [file pone.0264260.s001.docx]

**Innovative Support for Patients with SARS-COV-2 Infections Registry (INSPIRE): a longitudinal study of the medium and long-term sequelae of SARS-CoV-2 infection**

**Protocol**

Version draft - July 30, 2021

| Sponsors: | Center for Disease Control and Prevention |
| --- | --- |
| Protocol contributors: | Robert Weinstein, MD (Rush University)  Graham Nichol, MD (University of Washington)  Matthew Thompson, MD (University of Washington)  Arjun Venkatesh, MD (Yale University)  Erica Spatz, MD (Yale University)  Michael Gotlieb, MD (Rush University)  Kelli O'Laughlin, MD (University of Washington)  Nikki Gentile, MD (University of Washington)  AnnaMarie Chang, MD (Thomas Jefferson University)  Ralph Wang, MD (University of California San Francisco)  Rob Rodriguez, MD (University of California San Francisco)  Joann Elmore, MD (University of California Los Angeles)  Ahamed Idris, MD (University of Texas Southwest)  Ryan Huebinger, MD (University of Texas Houston)  Mandy Hill, MD (University of Texas Houston)  Ahamed Idris, MD (University of Texas Southwestern) |
| Trial identifiers: | ClinicalTrials.gov Identifier: NCT04610515  Other Study ID Numbers: 75D30120C08008 |

**REVISION HISTORY**

| Version | Date | Amendment Text | Description |
| --- | --- | --- | --- |

Revision history is listed in Appendix A on page 2.

**Table of Contents**

[1. TRIAL SUMMARY 5](#_Toc1)

[2. INTRODUCTION 7](#_Toc2)

[2.1. Background and rationale 7](#_Toc3)

[2.2. Objectives 7](#_Toc4)

[2.3. Trial design 8](#_Toc5)

[3. METHODS 8](#_Toc6)

[3.1. Study setting 8](#_Toc7)

[3.2. Eligibility criteria 8](#_Toc8)

[3.3. Recruitment 10](#_Toc9)

[3.4. Interventions 10](#_Toc10)

[3.4.1. Intervention description 10](#_Toc11)

[3.5. Outcomes 10](#_Toc12)

[3.6. Data collection 12](#_Toc13)

[3.6.1. Trial procedures and evaluations 12](#_Toc14)

[3.7. Human subjects considerations 13](#_Toc15)

[3.8. Sample size 14](#_Toc16)

[3.9. Statistical methods 15](#_Toc17)

[3.9.1. Outcomes 15](#_Toc18)

[3.10. Data management 16](#_Toc19)

[3.11. Data monitoring 16](#_Toc20)

[3.11.1. Interim analysis 16](#_Toc21)

[3.12. Reporting 16](#_Toc22)

[3.13. Research partnership 16](#_Toc23)

[3.14. Participant timeline 17](#_Toc24)

[3.15. Allocation 17](#_Toc25)

[3.16. Blinding (masking) 17](#_Toc26)

[3.16.1. Blinding mechanism 17](#_Toc27)

[3.17. Safety/harms 18](#_Toc28)

[3.18. Auditing 18](#_Toc29)

[4. ETHICS AND DISSEMINATION 18](#_Toc30)

[4.1. Research ethics approval 18](#_Toc31)

[4.2. Protocol amendments 18](#_Toc32)

[4.3. Informed consent process 18](#_Toc33)

[4.4. Confidentiality 18](#_Toc34)

[4.5. Declaration of interests 19](#_Toc35)

[4.6. Access to data 20](#_Toc36)

[4.7. Dissemination policy 20](#_Toc37)

[4.7.1. Trial results 20](#_Toc38)

[5. STUDY ADMINISTRATION 20](#_Toc39)

[5.1. Key contacts 20](#_Toc40)

[5.2. Funders 21](#_Toc41)

[5.3. Roles and responsibilities 21](#_Toc42)

[5.3.1. Protocol contributors 21](#_Toc43)

[5.3.2. Sponsor and funder 21](#_Toc44)

[5.3.3. Trial committees 21](#_Toc45)

[6. APPENDICES 21](#_Toc46)

[6.1. Informed consent materials 21](#_Toc47)

# 1. TRIAL SUMMARY

World Health Organization Registration Data Set

| Title | Innovative Support for Patients with SARS-COV-2 Infections Registry (INSPIRE): a longitudinal study of the medium and long-term sequelae of SARS-CoV-2 infection |
| --- | --- |
| Primary registry and trial identifying number | ClinicalTrials.gov Identifier: NCT04610515 |
| Secondary identifying numbers | 75D30120C08008 |
| Sources of monetary or material support | Centers for Disease Control and Prevention Financial and other support |
| Primary sponsor | Center for Disease Control and Prevention |
| Secondary sponsors (if any) | Not applicable |
| Central contact | Bala Hota, MD  Rush University  Bala_Hota@rush.edu |
| Study officials/Investigators | **Rush University, Administrative Core & Enrolling Site**  **Core Investigators:** Bala Hota, MD; Robert A. Weinstein, MD  **Core research team:** Katherine Koo, MS  **Site Investigators:** Michael Gottlieb, MD, Site Principal Investigator  **Site research team:** Michelle Santangelo, MPH  **Yale University, Analytic Core & Enrolling Site**  **Core Investigators:** Arjun Venkatesh, MD, MBA, MHS; Erica Spatz MD, MHS; Andrew Ulrich, MD  **Core research team:** Zhenqiu Lin, PhD; Shu-Xia Li, PhD; Huihui Yu, PhD; Mengni Liu, MS; Jeremiah Kinsman, MPH  **Site Investigators:** Arjun Venkatesh, MD, MBA, MHS; Erica Spatz MD, MPH; Andrew Ulrich, MD  **Site research team:** Jeremiah Kinsman, MPH; Michelle Opare, BS  **University of Washington, Clinical Core & Enrolling Site**   \| **Core Investigators:** Graham Nichol, MD, Principal Investigator; Matthew Thompson, MD, MPH, DPhil, Principal Investigator  **Core research team:** Jill Anderson, BSN, RN, Clinical Core Program Manager; Kari Black, BA, Grant & Finance Manager; Dana Morse BSN, Research Coordinator; Anoushka Fernandes BSc, Research Assistant.  **Site Investigators:** Kelli N. O’Laughlin, MD, MPH, Site Principal Investigator; Nikki Gentile, MD, PhD, Co-Investigator; Kari Stephens, PhD, Co-Investigator  **Site research team:** Rachel E. Geyer, MPH, Research Coordinator; Victoria Lyon, MPH, Program Manager; Sophie C. Morse, BA, BS, Research Assistant; Karen Adams, BA, Regulatory Specialist; Michael Willis, AS, BSHS, Research Assistant \| \| --- \|   **Thomas Jefferson University, Enrolling Site**  **Site Investigators:** Anna Marie Chang, MD, MSCE, Benjamin Slovis, MD, MA  **Site research team:** Morgan Kelly, BS, Alaina Hunt, BA, Kyle Norton, BA, Mubazar Ishfaq, BA, Paavali Hannikainen, BS, Melanie Chalfin, BA, Lindsey Shughart, Hailey Shughart, BA, Nicole Renzi, RN  **University of Texas Health Science Center at Houston, Enrolling Site**  **Site Investigators:** Mandy Hill, DrPH, MPH, Site Co-Principal Investigator; Ryan Huebinger Site, MD, Co-Principal Investigator; Summer Chavez, DO, MPH, MPM, Site Co-Investigator  **Site research team:** Elizabeth Vidales MD, MPH; Leslie Johnson BS  **University of Texas Southwestern Medical Center, Enrolling Site**  **Site Investigators:** Ahamed H. Idris, MD, PI; Samuel McDonald, MD, Co-I  **Site research team:** Paula Arellano-Cruz, Research Coordinator; David Gallegos, Research Associate  **University of California, Los Angeles, Enrolling Site**  **Site Investigators:** Joann Elmore, MD, MPH (site PI), Lauren Wisk, PhD  **Site research team:** Raul Moreno, BA, Dayna Clayton, BA, Annie Lee, PhD, Michelle L’Hommedieu, PhD, Chris Chandler, BA  **University of California, San Francisco, Enrolling Site**  **Site Investigators:** Robert Rodriguez, MD; Ralph C. Wang, MD, MAS; Juan Carlos Montoy, MD, PhD  **Site research team:** Robin Kemball, MPH; Virginia Chan, Cecilia Lara Chavez, Angela Wong |
| Brief title | INSPIRE Registry |
| Acronym | Innovative Support for Patients with SARS-COV-2 Infections (COVID-19) Registry (INSPIRE) |
| Countries of recruitment | USA |
| Condition(s) or focus of study | SARS-COV-2-2 infection |
| Interventions | Not applicable |
| Key eligibility criteria | Age eligibility: 18 years or older |
| Study design | Allocation: N/A  Primary purpose: Other  Phase: Phase 3 |
| Masking | Not applicable |
| Date of enrollment | November 1, 2020 |
| Target sample size | 3600 |
| Recruitment status | Recruiting |
| Primary outcomes | To understand the medium- and long-term sequelae of symptomatic SARS-CoV-2 infection |
| Secondary outcomes | To describe predictors of sequelae as reported by individuals and as recorded in their EMR, and to assess the intersection of long-term sequelae of COVID-19 with other previously defined syndromes with overlapping features such as myalgic encephalomyelitis/chronic fatigue syndrome (ME/CFS) |

# 2. INTRODUCTION

## 2.1. Background and rationale

The COVID-19 pandemic is associated with considerable morbidity and mortality. As of May 2021, > 32.6 million COVID-19 cases and > 580,000 attributed deaths have been detected in the USA [[1]](https://www.spiritprotocol.com/api/docx/https://paperpile.com/c/nKRweq/D7DYc). Globally, > 160 million COVID-19 cases and > 3.3 million attributed deaths have been reported [[2]](https://www.spiritprotocol.com/api/docx/https://paperpile.com/c/nKRweq/0puKg). The clinical course of acute COVID-19 is well-described [[3–7]](https://www.spiritprotocol.com/api/docx/https://paperpile.com/c/nKRweq/lU1D+t8iR+K9mW+Mq1f+OJ8y). Post-acute COVID-19 is defined as persistence of symptoms or development of sequelae after 3 or 4 weeks from the onset of acute symptoms of COVID-19 [[8–10]](https://www.spiritprotocol.com/api/docx/https://paperpile.com/c/nKRweq/XHsQi+bmgL6+etRcB). This definition is further divided into subacute or ongoing symptomatic COVID-19, including symptoms from 4-12 weeks beyond acute COVID-19, and a chronic or post-COVID-19 syndrome, which includes symptoms persisting or present beyond 12 weeks of acute COVID-19 not attributable to alternative diagnoses [[8,11]](https://www.spiritprotocol.com/api/docx/https://paperpile.com/c/nKRweq/XHsQi+1Igm8). Information on post-acute COVID-19 and long-term sequelae of SARS-CoV-2 infection is only recently emerging [[12–15]](https://www.spiritprotocol.com/api/docx/https://paperpile.com/c/nKRweq/DBuqU+NqrkR+3wwBG+PRH0f).

To characterize post-COVID-19 syndromes better, there is an urgent need for broader representation in study population selection to allow for representativeness and generalizability, to set objective outcomes that are not limited to symptoms but include illness events and clinical events, and to include SARS-CoV-2 negative individuals to ensure other effects of the pandemic are considered in the analysis (e.g. impact on livelihoods, mental health, food security, and mobility) [[16]](https://www.spiritprotocol.com/api/docx/https://paperpile.com/c/nKRweq/38UeS). Such work will help clinicians know what post-infectious sequelae to expect and who is at increased risk, will help ensure research findings can be compared across studies, and will move us towards addressing critical health care response needs.

To accelerate research during this critical time in the global COVID-19 pandemic, our research consortium designed a prospective longitudinal study to use patient-reported information linked to real-world data through the Innovative Support for Patients With SARS-COV-2 Infections Registry (INSPIRE) hosted on a secure online platform (Hugo, Hugo Health LLC, Guilford, CT) which imports health information from various sources with permission from participants. In this study, researchers follow a sample of individuals under investigation for SARS-CoV-2 over time, to collect patient-reported outcomes, interactions with the medical system (i.e., clinic visits, hospitalizations, laboratory test and medications prescribed) and outcomes of care as reported in the electronic medical record (EMR).

## 2.2. Objectives

Our objectives are to understand the medium- and long-term sequelae of symptomatic SARS-CoV-2 infection, to describe predictors of sequelae as reported by individuals and as recorded in their EMR, and to assess the intersection of long-term sequelae of COVID-19 with other previously defined syndromes with overlapping features such as myalgic encephalomyelitis/chronic fatigue syndrome (ME/CFS).

## 2.3. Trial design

This is a prospective, multicenter, longitudinal cohort study of individuals with acute symptoms consistent with SARS-CoV-2, including those with positive and negative diagnostic SARS-CoV-2 tests to compare those with and without SARS-CoV-2 infection (ClinicalTrials.gov Identifier: NCT04610515) [[17]](https://www.spiritprotocol.com/api/docx/https://paperpile.com/c/nKRweq/WTVFx).

# 3. METHODS

## 3.1. Study setting

Participants are enrolled from any of eight regions across the United States led by investigators at Rush University (Chicago, Illinois), Yale University (New Haven, Connecticut), the University of Washington (Seattle, Washington), Thomas Jefferson University (Philadelphia, Pennsylvania), the University of Texas Southwestern (Dallas, Texas), the University of Texas, Houston (Houston, Texas), the University of California, San Francisco (San Francisco, California) and the University of California, Los Angeles (Los Angeles County, California). The recruitment areas vary in terms of the population served to allow for ethnic and geographic diversity among study participants. As enrollment is through the internet and can be completed without assistance of research staff support, participants include those who have never been to the clinic or hospital for their symptoms and others who have been to the emergency department or hospitalized. Additionally, participants with any digital health portal can enroll in this study; participants’ health portals need not be those directly linked to the academic institutions listed above.

## 3.2. Eligibility criteria

This study includes adult patients who are under clinical investigation for possible SARS-CoV-2 infection and who meet inclusion criteria outlined in Table 1. We include individuals with symptomatic presentation to determine comparative frequency of outcomes amongst symptomatic individuals with and without SARS-CoV-2 infection: symptomatic individuals presenting with covid-like illness who test negative will act as controls for symptomatic individuals who have positive tests for SARS-CoV-2. Asymptomatic individuals are not eligible, given the anticipated lower rates of outcomes among asymptomatic individuals who test positive for SARS-CoV-2, and the significant heterogeneity in reasons for testing in asymptomatic individuals, such as screening for social, educational, occupational reasons and prior to routine clinical procedures. Individuals who self-report symptoms suggestive of acute SARS-CoV-2 infection, and are tested for SARS-CoV-2 within the last 42 days, are eligible to participate. From study initiation through the summer of 2021, symptoms of acute SARS-CoV-2 was defined using the COVID-19 clinical criteria case definition (Table 2) [[18]](https://paperpile.com/c/nKRweq/MqHQg); beginning summer of 2021, in order to capture less symptomatic individuals, the inclusion criteria were revised to require only one symptom among those listed in Table 2. Individuals for whom a SARS-CoV-2 test result cannot be confirmed are not eligible to participate in the study. Efforts are made to recruit participants from across the spectrum of COVID-19 illness severity, including individuals from outpatient (e.g., drive through testing with self-reported symptoms) to inpatient settings, and those cared for in intensive care units. We seek to enroll using a 3:1 case/control ratio to oversample those who are positive for SARS-CoV-2 on testing, while still ensuring an adequate control cohort for comparison.

**Table 1: Inclusion and exclusion criteria**

Inclusion criteria:

1. Fluent in English or Spanish
2. Age 18 years or older
3. Self-reported symptom suggestive of acute SARS-CoV-2 infection [[19]](https://www.spiritprotocol.com/api/docx/https://paperpile.com/c/nKRweq/KvIKQ)
4. Tested for SARS-CoV-2 with any FDA-approved or authorized viral test (i.e., nucleic acid amplification test or antigen test [[20]](https://www.spiritprotocol.com/api/docx/https://paperpile.com/c/nKRweq/mGkhJ)) within 42 days of enrollment

Exclusion criteria:

1. Unable to provide informed consent
2. Study team unable to confirm the result of a diagnostic test for SARS-CoV-2
3. Does not have access to a hand-held device or computer that would allow for digital participation in the study
4. Lawfully imprisoned while participating in the study

**Table 2: COVID-19 clinical criteria case definition (August 2020)** [[18]](https://www.spiritprotocol.com/api/docx/https://paperpile.com/c/nKRweq/MqHQg)

*In the absence of a more likely diagnosis,*

At least TWO of the following symptoms:

- Fever (measured or subjective)
- Chills
- Rigors
- Myalgia
- Headache
- Sore throat
- Nausea or vomiting
- Diarrhea
- Fatigue
- Congestion or runny nose

Or any ONE of the following symptoms:

- Cough
- Shortness of breath
- Difficulty breathing
- New olfactory disorder
- New taste disorder

Or severe respiratory illness with at least ONE of the following:

- Clinical or radiographic evidence of pneumonia
- Acute respiratory distress syndrome (ARDS)

## 3.3. Recruitment

Participant Identification and Enrollment

Methods used to recruit potentially eligible participants vary by site, although each site applies the same eligibility criteria described in Table 1. Most sites screen for eligible participants among those tested for SARS-CoV-2 infection. With this process in place, we seek to enroll participants as close to their initial date of SARS-CoV-2 testing as possible. Identification and enrollment methods include: i) a member of the study team accesses the EMR to screen for potentially eligible individuals based on SARS-CoV-2 testing and reason for testing and contact eligible individuals, ii) participants learn of the study from a poster, brochure, or social media and can enroll directly through the online portal, iii) research staff reach out to potentially eligible individuals in-person, over the phone, or by text or e-mail to offer and facilitate enrollment (e.g., contact information obtained from organizations including city and state agencies conducting SARS-CoV-2 testing). The method or combinations of methods used at each site are based on local IRB approval and practical considerations. Though minor differences exist across sites, the study eligibility criteria, online enrollment, and data collection methods are identical, which allows for compilation and comparison of data across sites.

## 3.4. Interventions

### 3.4.1. Intervention description

This is a prospective, observational study without an intervention.

## 3.5. Outcomes

Patient reported outcomes include self-reported disease-specific and generic health measures (Appendix A) [[21–23]](https://www.spiritprotocol.com/api/docx/https://paperpile.com/c/nKRweq/VLC3Q+9tpIp+1b0NF). Health care utilization and clinical events are extracted from the EMR data via Hugo as well as collected via patient self-report, ensuring uniform variable definitions across participating sites.

Self-report of symptoms suggestive of SARS-CoV-2 are assessed using questions derived from the Centers for Disease Control and Prevention (CDC) Person Under Investigation for SARS-CoV-2 survey (Appendix A) [[24]](https://www.spiritprotocol.com/api/docx/https://paperpile.com/c/nKRweq/QL4WH). This is not scored and has no predefined cutoff for the likelihood of COVID-19 illness.

Generic physical and mental health is assessed using the PROMIS^®^-29 [[25]](https://www.spiritprotocol.com/api/docx/https://paperpile.com/c/nKRweq/sSZ6k). This measure assesses pain intensity using a single 0-10 numeric rating item, and seven health domains (physical function, fatigue, pain interference, depressive symptoms, anxiety, ability to participate in social roles and activities, and sleep disturbance) using five response options per domain ranging from “not at all” to “very much”. In prior studies, this measure exhibits high reliability and validity, correlating well with other physical and mental health surveys as well as with chronic disease. The PROMIS^®^ instrument assesses health-related quality of life over the past seven days, except for two domains (physical function and ability to participate in social roles and activities) which do not specify a timeframe. Raw PROMIS^®^-29 scores are re-scaled from raw scores of 8 (worst) to 40 (best) into standardized T-score with a mean of 50 and standard deviation (SD) of 10. A higher PROMIS^®^ T-score represents more of the concept being measured. For negatively worded concepts like Anxiety, a higher score is worse. For positively worded concepts like Physical Function-Mobility, a higher score is better. Version 2.1 of the PROMIS^®^-29, which will be used in the present study, is rescaled into a generic, societal, preference-based summary score [[26]](https://www.spiritprotocol.com/api/docx/https://paperpile.com/c/nKRweq/smMJA). This is based on PROMIS^®^ scores for Cognitive Function, Abilities, Depression, Fatigue, Pain Interference, Physical Function, Sleep Disturbance, and Ability to Participate in Social Roles and Activities. It is scaled from 0 (equal to death) to 1 (equal to perfect health). Version 2.0 PROMIS^®^ scores can also be used to estimate a Health Utility Index Mark 3 preference score [[27]](https://www.spiritprotocol.com/api/docx/https://paperpile.com/c/nKRweq/0S9Lw). In other settings, differences of 0.03 to 0.1 in this preference score have been interpreted as being clinically important [[28–30]](https://www.spiritprotocol.com/api/docx/https://paperpile.com/c/nKRweq/Iu5iR+gmyam+fWtpT). A cutoff of 0.7 is used to determine severe impairment [[31]](https://www.spiritprotocol.com/api/docx/https://paperpile.com/c/nKRweq/wG4sA).

Cognition is assessed using the survey questions from the Patient-Reported Outcomes Measurement Information System (PROMIS^®^) Cognitive SF 8 [[32]](https://www.spiritprotocol.com/api/docx/https://paperpile.com/c/nKRweq/MUfAZ). Raw PROMIS^®^ Cognitive scores are re-scaled from raw scores of 8 (worst) to 40 (best) into standardized T-scores with a mean of 50 and standard deviation (SD) of 10. A higher score is interpreted as indicating greater cognition.

Health care process measures are assessed as ambulatory care and/or emergency department (ED) visits for symptoms related to COVID-19 illness as well as hospitalization (admitted to hospital overnight during study follow-up) as determined by data from the EMR.

Hospital-free and intensive care unit (ICU)-free survival are assessed as determined by data from the EMR [[33]](https://www.spiritprotocol.com/api/docx/https://paperpile.com/c/nKRweq/7mLIq). Hospital-free survival is survival without any hospitalizations and ICU-free survival is survival without time spent in the ICU.

Additional outcomes  assessed include post-infectious sequelae (e.g., dyspnea, cough) [[34–36]](https://www.spiritprotocol.com/api/docx/https://paperpile.com/c/nKRweq/crjeV+MWglS+v2y9g), post-traumatic stress disorder (PC-PTSD-5) [[37]](https://www.spiritprotocol.com/api/docx/https://paperpile.com/c/nKRweq/dFlhd), and exercise (exercise vital sign; 2 question survey to assess habitual physical activity) [[38–40]](https://www.spiritprotocol.com/api/docx/https://paperpile.com/c/nKRweq/E8xYP+3XcZq+NP9GE), using previously validated questionnaires .

Social determinants of health (e.g., housing, available social services, geographical location, and education) are assessed using a previously validated questionnaire [[41]](https://www.spiritprotocol.com/api/docx/https://paperpile.com/c/nKRweq/tBxfV).

Work and activity status are assessed using questions about returning to work, missed work and activity level (Appendix A).

Myalgic encephalomyelitis/chronic fatigue syndrome (ME/CFS) is assessed using the CDC Short Symptom Screener (Appendix A), following 2015 Institute of Medicine diagnostic criteria (Table 3) [[42]](https://www.spiritprotocol.com/api/docx/https://paperpile.com/c/nKRweq/JxTww). While the study design will allow for assessment of a range of clinical outcomes as well as correlation with various clinical syndromes, recognizing that early reports of post-COVID sequelae may overlap with ME/CFS, this syndrome is included as one of the specific outcomes assessed.

**Table 3. Diagnostic Criteria for Myalgic Encephalomyelitis/Chronic Fatigue Syndrome**

| Patients must have all four criteria:   1. A substantial reduction or impairment in the ability to engage in pre-illness levels of activity (occupational, educational, social, or personal life) that: a) lasts for more than 6 months, and b) is accompanied by fatigue that is often profound, of new onset (not lifelong), not the result of ongoing or unusual excessive exertion, and not substantially alleviated by rest. 2. Post-exertional malaise (defined as worsening of symptoms after physical, mental or emotional exertion that would not have caused a problem before the illness). 3. Unrefreshing sleep (defined as not feeling better or less tired even after a full night of sleep despite the absence of specific, objective sleep alterations). 4. At least one of the following two symptoms:    1. Cognitive impairment (defined as problems with thinking, memory, executive function, and information processing, as well as attention deficit and impaired psychomotor functions. These can be exacerbated by exertion, effort, prolonged upright posture, stress, or time pressure, and may have serious consequences on a patient’s ability to maintain a job or attend school full time).    2. Orthostatic intolerance (defined as a worsening of symptoms upon assuming and maintaining upright posture as measured by objective heart rate and blood pressure abnormalities during standing, bedside orthostatic vital signs, or head-up tilt testing. Orthostatic symptoms can include lightheadedness, fainting, increased fatigue, cognitive worsening, headaches, or nausea that are worsened with upright posture (either standing or sitting) during day-to-day life and are improved (though not necessarily fully resolved) with lying down). |
| --- |

## 3.6. Data collection

### 3.6.1. Trial procedures and evaluations

Results for a SARS-CoV-2 viral test (i.e., nucleic acid amplification test or antigen test) are confirmed by the research staff either through visualizing the result in the EMR or by reviewing an image of the test result sent to the research staff by the participant. After an eligible patient enrolls in the study, a combination of self-reported information and information generated from the patient’s own health information is connected to the Hugo platform, collected, and sent to the study site over the 18-month follow-up period (Appendix A).

Self-reported data: Using Hugo, surveys including the variables outlined above are sent by electronic mail or text to participants at the research site every 3 months throughout the follow-up period. These responses are sent through the Hugo platform and then shared with the study team. Subjects use their personal smartphone, tablet, computer, or other electronic device to connect to the internet and answer surveys that ascertain their symptoms, health care use, health care experience, and physical, mental, and social health over an 18-month period. Responses sent by participants are encrypted. To minimize participant burden, each data collection episode is designed to take approximately 15 minutes or less to complete.

Patient-Centric Data Sharing: Participants connect their own health system portal account with the Hugo platform at enrollment (Figure 1). Hugo is a web-based platform used to longitudinally collect data for this study. The Hugo platform gives patients the ability to collect and maintain their personal health records in a centralized, cloud-based account. Participants will create a Hugo account and connect the health system portal accounts they choose to connect with the Hugo platform. This may include patient portals from healthcare systems, pharmacies, laboratories, and insurers. Individuals direct Hugo to share their health records with the research team according to the terms in the informed consent. This information is sent from Hugo to the research analytic core and stored in accordance with their institutional policies. The specific portal-related information available through Hugo in use for INSPIRE includes hospital and clinical patient portals yielding current data on medications, appointments and visits, test orders and results, clinical notes, problems, diagnoses, vital signs, demographics, and immunizations. After the study, participants can maintain their Hugo account or opt to delete their account and data.

Participants can connect their health system(s) portal accounts with Hugo at set up but may need assistance if they have technical issues or if they do not complete this initial step at enrollment. Once participants create an account in Hugo and link their portals, no additional actions are required to stream data into Hugo. Technical support from the enrolling site or clinical core is provided to resolve any difficulties setting up an account.

During the study, periodically Hugo downloads identifiable data outlined in the IRB protocol and consent form. Research sites have access to site-specific dashboards to track enrollment, identify which data sources are connected, and to monitor survey responses. Deidentified, individual-level data are sent from Hugo to the analytic team periodically for quality assurance and analysis.

## 3.7. Human subjects considerations

Participants are eligible to receive a small incentive for completion of each periodic questionnaire; the total value will be $100 over the course of the study. Researchers will not provide any information gathered through the study to clinicians engaged in treating the patient. Patients will be informed and reminded that their responses will not be provided to their healthcare team, both at the beginning of the study during the consent process and throughout the study on the regular questionnaires.

## 3.8. Sample size

Our target enrollment is 3,600 people with SARS-CoV-2 infection confirmed by a positive SARS-CoV-2 viral test (i.e., nucleic acid amplification test or antigen test) and 1,200 people with a negative SARS-CoV-2 viral test. We expect that the age distribution of enrolled subjects will represent that of patients tested in each site. Four of the sites (Yale, Jefferson, Rush and UW) draw on smaller catchment populations so have planned enrollment of 400 subjects per site (300 individuals with SARS-CoV-2 and 100 individuals without SARS-CoV-2). Four sites (UT Southwestern, UT Houston, University of California, San Francisco, and University of California, Los Angeles) have larger catchment populations so have planned enrollment of 800 subjects per site (600 individuals with SARS-CoV-2 and 200 individuals without SARS-CoV-2).

We estimated the power to detect relative differences in outcome rates between those who are symptomatic and test positive for SARS-CoV-2 as compared to those who are symptomatic but test negative for SARS-CoV-2. These power calculations are agnostic to the outcome of interest but based on the aim to examine relative differences in long-term outcomes between individuals with and without SARS-CoV-2 based on and between age strata. For comparison of ME/CFS incidence in SARS-CoV-2 infected vs uninfected, power calculations are based on the null hypothesis that there is no difference between individuals with and without SARS-CoV-2 in the outcome rate.

The assumptions used to generate these power calculations include both elements outside the study’s team’s control (e.g., baseline outcome rate in individuals without SARS-CoV-2) as well as elements amenable to changes in study design (e.g., individuals with SARS-CoV-2 strata group size).  Assumptions include:

- 3,600 individuals with SARS-CoV-2 and 1,200 individuals without SARS-CoV-2
- Baseline outcome rate in individuals without SARS-CoV-2: 2.5% with the contingency that the baseline outcome rate may vary between age strata (18-40 years, 41-64 years, ≥ 65 years)
- Outcome rate in individuals with SARS-CoV-2: presented as scenarios based on absolute or relative differences from baseline outcome rate in individuals without SARS-CoV-2. Also presented under the likely scenario that outcome rates vary between age strata
- Alpha = 0.05 (fixed)

Under conservative assumptions, the planned sample has 97.8% power to detect an absolute outcome rate difference of 2.5% between individuals with versus without SARS-CoV-2 infection. The power to detect a relative difference in outcome rates is highly sensitive to both the actual baseline outcome rate as well as variation in baseline outcome rates between age strata. The study would have adequate power (conventionally defined as 0.8) to detect a difference as small as 5% in outcomes with 1,224 total participants (25.5% of total planned enrollment) enrolled in a 3:1 ratio, or 918 COVID+ and 306 COVID negative (Table 4).

**Table 4. Event Rate Differences Between Groups**

|  | **0.07 vs 0.05** | | | **0.10 vs 0.05** | | | **0.15 vs 0.05** | | | **0.20 vs. 0.05** | | |
| --- | --- | --- | --- | --- | --- | --- | --- | --- | --- | --- | --- | --- |
| **Power** | **Total  N** | **COVID**  **(+)** | **COVID**  **(-)** | **Total N** | **COVID**  **(+)** | **COVID**  **(-)** | **Total  N** | **COVID**  **(+)** | **COVID**  **(-)** | **Total N** | **COVID**  **(+)** | **COVID**  **(-)** |
| 0.9 | 8012 | 6009 | 2003 | 1592 | 1194 | 398 | 516 | 387 | 129 | 180 | 135 | 45 |
| 0.8 | 6076 | 4557 | 1519 | 1224 | 918 | 306 | 404 | 303 | 101 | 144 | 108 | 36 |
| 0.7 | 4844 | 3633 | 1211 | 988 | 741 | 247 | 332 | 249 | 83 | 120 | 90 | 30 |
| 0.6 | 3904 | 2928 | 976 | 808 | 606 | 202 | 272 | 204 | 68 | 100 | 75 | 25 |

## 3.9. Statistical methods

### 3.9.1. Outcomes

Statistical analyses will describe disease course and outcomes, including the characterization of specific symptoms and duration of symptoms, health care utilization (emergency department, hospitalization, post-acute care) with clinical (morbidity/mortality) and patient-reported health status outcomes as well as recovery (early and late disease sequelae). Baseline and demographic characteristics will be summarized by descriptive summaries (e.g., means and standard deviations for continuous variables such as age and percentages for categorical variables such as gender, medians, and quartiles for skewed data). Analyses will:

- Compare health status at baseline and follow-up between persons in the same age group who test SARS-CoV-2 positive and negative at initial test.
- Characterize health care utilization (ambulatory and ED visits, hospitalizations, post-acute care, telehealth visits) among SARS-CoV-2 positive participants by age and compare these to SARS-CoV-2 negative participants.
- Characterize and compare health outcomes by age and SARS-CoV-2 status: emergency or ambulatory care, admission to hospital); ICU-free survival;^16^ hospital-free survival; and subsequent patient-reported health status (cognition; physical health; mental health; and return to work).

We will use statistical modeling to estimate the association between key covariates and outcomes, including evaluating interactions by age. We plan to use survival analysis techniques to analyze time to outcome events, logistic regression for binary outcomes (e.g., hospitalization versus no hospitalization), Poisson or Cox regression for count data (e.g., number of hospitalizations over time), and linear regression models for continuous outcomes (e.g., PROMIS-29). Multiple imputation will be considered to handle missing covariate and outcome data in these analyses [[43]](https://www.spiritprotocol.com/api/docx/https://paperpile.com/c/nKRweq/3dpny). Chained equations will be used to impute each variable with missing data. Sensitivity analyses will be conducted using missing categories for covariates and including only people with non-missing outcome information.

We will use statistical analyses to address the risk of ME/CFS and other health conditions in those with versus without SARS-CoV-2 infection as risk difference or risk ratios or odds ratios, as appropriate. We will also adjust for additional patient-level factors of interest, including age, sex, race/ethnicity, income, and presence of specific underlying conditions such as hypertension and diabetes. We anticipate that as more is learned about the clinical courses of persons with symptomatic SARS-CoV-2 infection, additional covariates of interest available from electronic record data will be assessed for their association with adverse outcomes.

## 3.10. Data management

Data is initially collected onto the HUGO HEALTH platform with participant permission. Intermittently, data will be pulled and shared with the analytic core at Yale University.

## 3.11. Data monitoring

### 3.11.1. Interim analysis

Periodic reports of de-identified data from participants at each site are exported from the Hugo data management system for monitoring and analysis by the study cores. These data will be used to monitor the adequacy of recruitment, with due consideration to the balance of subjects with positive versus negative tests as well as their age distribution, and completeness of follow-up questionnaires.

## 3.12. Reporting

The results of this study will be reported using the STrengthening the Reporting of OBservational studies in Epidemiology (STROBE) guidelines for reporting observational studies [[44]](https://www.spiritprotocol.com/api/docx/https://paperpile.com/c/nKRweq/ohVFp). We intend to disseminate the results as rapidly as possible to help contribute to the COVID-19 response.

## 3.13. Research partnership

This research will benefit from the contributions of a Patient Advisory Board. This group provides valuable perspective and guides our research team on issues pertaining to participant engagement, survey data collection, and will assist with interpretation of study findings and communicating results to the general population. Additionally, this study is conducted in collaboration with colleagues from the CDC. Scientists from the CDC assisted with study design and will remain engaged as we evaluate and disseminate the research findings.

## 3.14. Participant timeline

Figure 1. Participant Timeline

|  | Enrolment | Post-enrollment |  |  |  |  |  | Close-out |
| --- | --- | --- | --- | --- | --- | --- | --- | --- |
| TIMEPOINT | 0 | 3 mos. | 6 mos. | 9 mos. | 12 mos. | 15 mos. | 18 mos. | tx |
| ENROLLMENT: |  |  |  |  |  |  |  |  |
| Eligibility screen | X |  |  |  |  |  |  |  |
| Informed consent | X |  |  |  |  |  |  |  |
| ASSESSMENTS: |  |  |  |  |  |  |  |  |
| Age, gender, symptoms of SARS-COV-2 infection, recent health care use, social determinants of health | X |  |  |  |  |  |  |  |
| Outpatient visit, emergency department visit, hospitalization, intensive care stay, death, physical and mental health (PROMIS-29), cognitive SF-8, hospital-free survival, intensive care-free survival, dyspnea, cough, exercise, social determinants of health, work and activity status, myalgic encephalomyelitis/chronic fatigue syndrome |  | X | X | X | X | X | X | X |
| Preexisting conditions |  | X | X | X | X |  |  | X |

## 3.15. Allocation

Not applicable.

## 3.16. Blinding (masking)

### 3.16.1. Blinding mechanism

Not Applicable.

## 3.17. Safety/harms

Study risks are related to invasion of privacy and potential breach in confidentiality. Data maintained at the research site will include minimum necessary information and will be secured according to institution standards. Data maintained by Hugo Health will be subject to their privacy security policies https://hugo.health/security), Privacy Notice (https://hugo.health/privacy-notice), Terms of Service (https://hugo.health/terms-of-service/). Participants may experience inconvenience of filling out electronic patient-reported outcome measure surveys, which should take up to 20 minutes for the longer surveys (at enrollment baseline), and 15 minutes for the weekly/monthly surveys.

## 3.18. Auditing

Not Applicable.

# 4. ETHICS AND DISSEMINATION

## 4.1. Research ethics approval

Ethics approval of this protocol has been obtained at each individual site including Rush University (protocol number: 20030902, approved 3/14/2020), Yale University (2000027976, approved 4/30/2020), the University of Washington (UW Human Subjects Division, STUDY00009920, approved 4/2/2020), Thomas Jefferson University (20p.1150, approved 1/21/2021), the University of Texas Southwestern Medical Center (STU 2020-1352, approved 2/3/2021), the University of Texas, Houston (HSC-MS-20-0981, approved 9/10/2020), the University of California, San Francisco (20-32222, approved 1/25/2021) and the University of California, Los Angeles (20-001683, approved 12/18/2020). The Yale University ethics approval includes the role as the analytic lead. Additionally, the Rush University ethics approval includes INSPIRE data storage on the Hugo platform and transfer of data to Rush for secure storage.

## 4.2. Protocol amendments

Listed in Appendix A on Page 2 (uploaded with manuscript submission as supported information).

## 4.3. Informed consent process

This study involves self-enrollment with an online consent process using an electronic consent form designed with easy-to-read language. The consent form is a click-through digital document where, after reading the document, the participant clicks to agree or disagree to study participation. Electronic consent occurs through the Hugo platform.

## 4.4. Confidentiality

Privacy concerns pertaining to the use of the Hugo platform will be directed to Hugo and their privacy policies.

Subjects may feel that participating in this study is an invasion of their privacy. Subjects choose to share their personal health records with the study team. This will be outlined in the consent form and explained during any phone-based communications. Some subjects may feel a violation of privacy if they are directly contacted by the study team and ask to participate in the study. We will explain how patient information is obtained and why the patient is being approached about this study.

Self-reports of information shared via the Hugo platform for research will also be stored in the patient’s Hugo account along with the patients PHR data. This information will remain in their account unless directed by the participant to delete it. This information is explained in the Hugo terms and services and in the study consent form.

## 4.5. Declaration of interests

The Innovative Support for Patients with SARS-COV-2 Infections (INSPIRE) Registry is funded by the Centers for Disease Control and Prevention (CDC), National Center of Immunization and Respiratory Diseases (NCIRD) (contract number: 75D30120C08008; co-PIs Bala Hota, MD, and Robert A. Weinstein, MD). Partners from the CDC assisted with study design, the preparation of this protocol manuscript and the decision to publish this manuscript. The findings and conclusions in this report are those of the authors and do not necessarily represent the official position of the Centers for Disease Control and Prevention (CDC). No others sponsors or funders (other than the named authors) played any role in the study design, data collection and analysis, decision to publish, or preparation of the manuscript.

Additionally, I read the journal's policy and the authors of this manuscript have the following financial disclosures: JGE is an Editor for UpToDate on topics related to SARS-CoV-2 (https://www.uptodate.com). GN receives salary support from Leonard A Cobb~ Medic One Foundation ([https://www.mediconefoundation.org/about/who-we-are/](https://www.spiritprotocol.com/api/docx/https://www.mediconefoundation.org/about/who-we-are/)), ZOLL Circulation Inc. (https://www.zoll.com/contact/careers-at-zoll/tms), General Electric Health Care Inc. ([https://www.gehealthcare.com/about/about-ge-healthcare-systems](https://www.spiritprotocol.com/api/docx/https://www.gehealthcare.com/about/about-ge-healthcare-systems)), Kestra Medical Technologies (https://kestramedical.com/), Abiomed Inc. ([https://www.abiomed.com/](https://www.spiritprotocol.com/api/docx/https://www.abiomed.com/)), CPR Therapeutics Inc. (https://www.cprtherapeutics.com/), Pagonia Medical Inc. (no related website), and Medical Developments International ([https://medicaldev.com/](https://www.spiritprotocol.com/api/docx/https://medicaldev.com/)). AHI participates in the National Emergency Cardiovascular Care Committee (Science Subcommittee) of the American Heart Association (https://www.heart.org/) and is an unpaid member of the Clinical Advisory Board for Stryker ([https://www.stryker.com/us/en/index.html](https://www.spiritprotocol.com/api/docx/https://www.stryker.com/us/en/index.html)). HMK received expenses and/or personal fees from UnitedHealth ([https://www.uhc.com/](https://www.spiritprotocol.com/api/docx/https://www.uhc.com/)), IBM Watson Health ([https://www.ibm.com/watson-health?cm_sp=Scheduler-_-CopyChng2-_-C](https://www.spiritprotocol.com/api/docx/https://www.ibm.com/watson-health?cm_sp=Scheduler-_-CopyChng2-_-C)), Element Science ([https://www.elementscience.com/](https://www.spiritprotocol.com/api/docx/https://www.elementscience.com/)), Aetna ([https://www.aetna.com/](https://www.spiritprotocol.com/api/docx/https://www.aetna.com/)), Facebook ([https://www.facebook.com/](https://www.spiritprotocol.com/api/docx/https://www.facebook.com/)), the Siegfried and Jensen Law Firm ([https://www.siegfriedandjensen.com/](https://www.spiritprotocol.com/api/docx/https://www.siegfriedandjensen.com/)), Arnold and Porter Law Firm ([https://www.theworldlawgroup.com/member-firms/arnold-porter](https://www.spiritprotocol.com/api/docx/https://www.theworldlawgroup.com/member-firms/arnold-porter)), Martin/Baughman Law Firm ([https://www.martinbaughman.com/practice-areas/](https://www.spiritprotocol.com/api/docx/https://www.martinbaughman.com/practice-areas/)), F-Prime ([https://fprimecapital.com/](https://www.spiritprotocol.com/api/docx/https://fprimecapital.com/)), and the National Center for Cardiovascular Diseases in Beijing (<http://english.pumc.edu.cn/centers/62.html>). He is a co-founder of Refactor Health ([https://www.refactorhealth.com/](https://www.spiritprotocol.com/api/docx/https://www.refactorhealth.com/)) and had grants and/or contracts from the Centers for Medicare & Medicaid Services ([https://www.cms.gov/](https://www.spiritprotocol.com/api/docx/https://www.cms.gov/)), Medtronic ([https://www.medtronic.com/us-en/index.html](https://www.spiritprotocol.com/api/docx/https://www.medtronic.com/us-en/index.html)), the U.S. Food and Drug Administration ([https://www.fda.gov/](https://www.spiritprotocol.com/api/docx/https://www.fda.gov/)), Johnson & Johnson ([https://www.jnj.com/](https://www.spiritprotocol.com/api/docx/https://www.jnj.com/)), and the Shenzhen Center for Health Information (<http://www.sz.gov.cn/en/>). MT provided consulting as a paid medical advisor to Visby Medical ([https://www.visbymedical.com/](https://www.spiritprotocol.com/api/docx/https://www.visbymedical.com/)) and Roche Molecular Diagnostics (https://diagnostics.roche.com/) which both produce laboratory tests for COVID-19. The authors not otherwise listed here have no financial disclosures.

**COMPETING INTERESTS**

I read the journal's policy and the authors of this manuscript have the following competing interests: HMK is co-founder for HugoHealth.

## 4.6. Access to data

The final data set will be accessible by study investigators, the CDC funders, and others as permitted contractually by the CDC.

## 4.7. Dissemination policy

### 4.7.1. Trial results

Research findings will be disseminated through presentations to international audiences as well as through publications in peer-reviewed journals.

# 5. STUDY ADMINISTRATION

## 5.1. Key contacts

**Central contact**

Bala Hota, MD

Rush University

Bala_Hota@rush.edu

**Sponsor**

Sharon Saydah

Center for Disease Control and Prevention

[zle0@cdc.gov](mailto:zle0@cdc.gov)

## 5.2. Funders

Centers for Disease Control and Prevention

## 5.3. Roles and responsibilities

### 5.3.1. Protocol contributors

Bala Hota, MD

Department of Medicine, Division of Infectious Diseases, Rush University Medical Center, Chicago, IL

Graham Nichol, MD, MPH

Departments of Medicine and Emergency Medicine, University of Washington, Seattle, WA

Robert A. Weinstein, MD

Department of Internal Medicine, Division of Infectious Diseases, Rush University Medical Center; Department of Medicine, Cook County Health, Chicago, IL

Matthew Thompson, MBChB, MPH, DPhil

Department of Family Medicine, University of Washington, Seattle, WA

Kelli N. O’Laughlin, MD, MPH

Departments of Emergency Medicine and Global Health, University of Washington, Seattle, WA

Michael Gottlieb, MD

Department of Emergency Medicine, Rush University Medical Center, Chicago, IL

Erica Spatz, MD, MHS

Department of Cardiovascular Medicine, Yale University, New Haven, CT

Arjun Venkatesh, MD, MBA, MHS

Department of Emergency Medicine, Yale University, New Haven, CT

Ian D. Plumb, MBBS, MSc

Centers for Disease Control and Prevention, Division of Foodborne, Waterborne and Environmental Diseases, Atlanta, GA

Anna Marie Chang, MD

Department of Emergency Medicine, Thomas Jefferson University, Philadelphia PA

Lauren E. Wisk, PhD

Department of Medicine, David Geffen School of Medicine at UCLA, Los Angeles, CA

Aron J. Hall, DVM, MSPH

Centers for Disease Control and Prevention, Division of Viral Diseases, Atlanta, GA

Ralph C. Wang, MD, MAS

Department of Emergency Medicine, University of California, San Francisco, CA

Kari A. Stephens, PhD

Department of Family Medicine, University of Washington, Seattle, WA

Ryan M. Huebinger, MD

Department of Emergency Medicine, Rush, Houston, TX

Samuel A. McDonald, MD, MS

Department of Emergency Medicine and Clinical Informatics Center, UT Southwestern, Dallas, TX

Nikki Gentile, MD, PhD

Department of Family Medicine, University of Washington, Seattle, WA

Benjamin H. Slovis MD MA

Department of Emergency Medicine, Thomas Jefferson University, Philadelphia, PA

Mandy Hill, DrPH, MPH

Department of Emergency Medicine, UTHealth McGovern Medical School, Houston, TX

Sharon Saydah, PhD

Centers for Disease Control and Prevention, Division of Viral Diseases, Respiratory Viruses Branch, Atlanta, GA

Ahamed H. Idris, MD

Departments of Emergency Medicine and Internal Medicine, UT Southwestern, Dallas, TX

Robert Rodriguez, MD

Department of Emergency Medicine, University of California San Francisco, San Francisco, CA

Harlan M. Krumholz, MD, SM

Section of Cardiovascular Medicine, Yale University, New Haven, CT

Joann G. Elmore, MD, MPH

Department of Medicine, David Geffen School of Medicine at UCLA, Los Angeles, CA

### 5.3.2. Sponsor and funder

The study sponsor and funders provided periodic advice about study design; collection, management, analysis, and interpretation of data; writing of the report; and the decision to submit the report for publication, but they do not have ultimate authority over any of these activities.

### 5.3.3. Trial committees

The administrative core (Rush) oversees the primary contract with the sponsor, subcontracts with the clinical core (University of Washington [UW]) and the analytic core (Yale University), and three vanguard enrolling sites (Rush, UW, Yale) as well as the data software vendor (Hugo Health LLC, Guilford, CT).

The clinical core (UW) oversees monitoring and improvement of enrollment and retention at all sites (above, Thomas Jefferson University, University of California San Francisco, University of California Los Angeles, University of Texas Southwestern, University of Texas Houston).

The analytic core (Yale) oversees data analysis and reporting.

The study has a decision-making executive committee, an advisory steering committee, and a publications committee.

# 6. APPENDICES

## 6.1. Informed consent materials

**UNIVERSITY OF WASHINGTON**

**Research Consent for the COVID INSPIRE Registry**

Researcher: Kelli O’Laughlin, MD, MPH, Emergency Medicine, University of Washington, 206-543-3240 INSPIREREGISTRY@UW.EDU

You are invited to take part in a research study at the University of Washington. We are bringing together people being tested for COVID-19 with researchers to learn as much as we can and as fast as we can so that we are better prepared for this and future health challenges.

Before you agree to participate in a research study called the COVID INSPIRE Registry, it is important that you understand what is involved and what will be done with the information you provide. This form contains answers to some of the questions you might have so you can decide whether or not to join the

study. Being in this study is voluntary. Please read this information carefully. At the end of this form, you will indicate if you would like to join this study.

**What is the purpose of this study?**

We are partnering with people who receive diagnostic testing for COVID-19 and asking them to join a research study. Our goal is to better understand the experience of adults who receive diagnostic testing and may or may not be diagnosed with COVID-19. This study will use electronic tools to quickly collect

and share health information for scientists to better understand the COVID-19 outbreak. Some traditional ways of collecting data can take much longer to analyze information and share knowledge with the public. This study will take place across the United States. Researchers at the University of Washington are enrolling people in the local area to join this study.

**Who should sign up for this study?**

We are asking adults who receive diagnostic testing for COVID-19 to sign up for this research study. We want to understand the experience of people who receive clinical testing for COVID-19 regardless of if you have a diagnosis of COVID-19 or if the test results come back negative.

Right now, we are asking people to join this study if they tested for COVID-19 and can share documentation of their result with the research team, through the personal health record portal linked to the study platform. Only individuals who receive testing are asked to sign up for this study.

If you are a friend or family member of someone who received testing, you can encourage them to join this study. You should only sign up for this study if you have access to a personal mobile smart phone, tablet, or computer with internet access.

**What will happen if I agree to join this study?**

If you agree to join this study, you will first provide your consent to participate below. This consent will also authorize Hugo Health to share the health information and survey responses you connect and provide in Hugo platform with the study team. If you volunteer for this study, the following activities will occur over the 18 month study period and will take place remotely.

The initial set up process outlined in #1-3 could take approximately an hour in total. You might do this set up at one time or over a few days.

**#1. Initial set up in the Hugo platform**: Using your own mobile device (smartphone or tablet) or computer with internet access, you will register for the Hugo platform and if you choose, download the app. Registration for Hugo will require you to enter basic information including first name, last name, email address, and you will be asked to choose a password. You will follow instructions in the Hugo platform to activate your account with Hugo.

**#2. Connect your health systems and start sharing data to the study:** In Hugo, you will link your patient portal accounts and this information will be shared with the research study as described below:

Hospitals, clinics, and pharmacies: In Hugo, you can select the systems where you have received care and enter your patient portal login and password. For this study, we are interested in information from health clinics, hospitals, and pharmacies where you receive care.

Insurer: If you have health insurance coverage, you will also link your account with the Hugo platform. Hugo can gather information from your claims data to provide information on health care you receive while participating in the study.

Personal device: You have the option to connect a personal device, such as an activity tracker (Fitbit, Apple watch, or other device type) to share activity information in the Hugo platform.

If you have questions about setting up any of your accounts, please reach out to the study team for help. The study coordinator will help you with the study information and be available to answer any questions related to data sharing.

After you link your accounts, the Hugo platform will pull together your personal health information on your behalf. Information will then be shared with the study team for the purposes of this research. Health information shared to the COVID INSPIRE Registry may include information such as medications, medical problems, allergies, medical procedures, clinical encounters, lab results, diagnoses, vital signs, immunizations, and possibly other data that becomes available. From your personal device(s), the data being shared may include sleep patterns, movement (steps per-day), weight and body mass index. Information from the Hugo platform will be continuously shared throughout the study duration.

**#3. Complete initial research questions:** After you set up your account in Hugo, you will be asked to answer initial questions about your health, your social situation, and how you feel. You can choose to answer these questions by email or text message. You will receive a secure link with these questions for your response. It will take about 20 minutes to answer this first set of questions.

**#4. Complete research questions over the 18 months study period:** You will continue to receive surveys that ask questions about your health status, symptoms, and access to care by text or email throughout the study. Each set of surveys you receive will take no more than 20 minutes to complete. After you complete the initial set of research questions when you sign up for this study, you will be asked to answer surveys 6 times over the next 18 months (once every 3 months).

**#5. Contact for Future Research:** One goal of this study is to store names and contact information of individuals interested in participating in future research opportunities related to COVID-19. This is an optional part of the study. If you agree to this part of the study, only the study team at the University of Washington will have access to personal identifiers and contact information to contact you for future research. In the case a study is designed in the future, the UW team may contact you to see if you are interested. We will not give names or any personal information to other researchers for this purpose. If you decide to take part in a future study, you will need to review and sign a separate consent form.

**Are there risks to participating in this study?**

The risks related to this study include risks of invasion of privacy and breach in confidentiality. In this study, you will share personal health information with researchers. There is a risk that someone might access that information improperly.  We explain how we will store and share information from this study with others, and how the data will be confidential below.

While participating in this study, you will be asked to fill out multiple surveys, which will take some time and may be inconvenient. Some of the surveys may include questions that you feel uncomfortable answering. If you feel uncomfortable answering any specific survey question, you will be able to skip these questions and continue with the rest of the survey questions.

**Are there benefits to participating in this study?**

You may not directly benefit from taking part in this study, but we hope that knowledge gained from this study may benefit others with COVID-19 in the future. This study allows you to join a community of people, participating together in generating knowledge about this condition. Our goal is to share information from scientific presentations and paper publications from this study also with study participants. This information would be shared through the Hugo platform.

**Will I receive any individual results?**

Researchers will not be watching or evaluating your symptoms as part of this study, including your responses to the questionnaires. None of the information collected in this study will be shared with your medical team. If at any point you begin to experience new symptoms, or any medical issues arise, please contact your doctor. In case of a life- threatening emergency, call 911 immediately.

**Will study data be kept confidential?**

To participate in this study, you will give permission to the Hugo system to collect personally identifiable information (like your name and where you go to the doctor) along with a large quantity of information about your health and care. The Hugo platform uses precautions, including industry standard encryption, to minimize privacy and security risks to your stored information. To learn more about Hugo’s security and privacy policies, please review their Terms of Service, and Privacy and Security Statements.

The Hugo system will share health information that you provide by connecting patient portals and completing surveys with the research team at University of Washington and Yale University. With this information, these researchers will have access to personal identifiers such as name, contact information, MRN, and DOB. Identifiers will be stored separate from other information about participants. The research team has strict protocols in place to control access to your data. Researchers will only be able to view the health data that you connect to the Hugo platform that you allow to be shared for this study. The researchers will not give your doctors any information from the questionnaires you fill out; that data will be kept confidential and will not be part of your medical record.

The information collected for this study will be kept for an indefinite period at the University of Washington and Yale University, under the responsibility of the study team.

For participants interested in being contacted for future research, the University of Washington will maintain a list of participants and their contact information for that purpose.

Government (Center for Disease Control and Prevention or the Office for Human Research Protections) or university staff sometimes review studies such as this one to make sure they are being done safely and legally.  If a review of this study takes place, your records may be examined.  The reviewers will protect your privacy.  The study records will not be used to put you at legal risk of harm.

If we publish any research or other documents based on data from this study, the research will never identify a participant by name.

We have a Certificate of Confidentiality from the federal Centers for Disease Control (CDC).  This helps us protect your privacy.  The Certificate means that we do not have to give out information, documents, or samples that could identify you even if we are asked to by a court of law.  We will use the Certificate to resist any demands for identifying information.

We can’t use the Certificate to withhold your research information if you give your written consent to give it to an insurer, employer, or other person.  Also, you or a member of your family can share information about yourself or your part in this research if you wish.

There are some limits to this protection. We will voluntarily provide the information to:

- a member of the federal government who needs it in order to audit or evaluate the research;
- individuals at the institution(s) conducting the research, the funding agency, and other groups involved in the research, if they need the information to make sure the research is being done correctly;
- the federal Food and Drug Administration (FDA), if required by the FDA;
- individuals who want to conduct secondary research if allowed by federal regulations and according to your consent for future research use as described in this form;
- appropriate authorities, if we learn of child abuse, elder abuse, or the intent to harm yourself or others.

The Certificate expires when the CDC funding for this study ends. Any data collected after expiration is not protected as described above.  Data collected prior to expiration will continue to be protected.

**How will data be shared with others?**

One goal of this study is to rapidly collect data on a large population of people who receive testing for COVID-19 to facilitate sharing of data for future research. For this reason, the information that we obtain will be shared with researchers for future undefined studies. When data is shared, it will be identified by a code and not by identifiers.

It is also possible that in the future we may want to use or share study information that might identify you. If we do, a review board will decide whether or not we need to get additional permission from you.

**Do I have to participate in the study?** Your participation in this research is completely voluntary.

**Are there costs to participate in this study?** There are no costs to participate in this study. If you choose to access the Hugo platform on your hand-held device and/or have surveys sent by text, please remember that data rates according to your cellular plan will apply.

**Will I be paid to participate in this study?** As a study participant, you will be paid $25 for the first survey. You will receive the following payments for completion of the other surveys:

Month 3:              $10

Month 6:              $10

Month 9:              $10

Month 12:            $10

Month 15:            $10

Month 18:            $25

**Who is funding this research?** The study team and/or the University of Washington is receiving financial support from the Centers for Disease Control and Prevention (CDC).

**Can I stop participating in this study if I change my mind?** You can stop participating in this study at

any time. If you wish to withdraw from the study, please email the study team at

INSPIREREGISTRY@UW.EDU. If you withdraw from the study, we will stop sending you information to complete, and no new health information about you will be shared with the study.

You can also remove your information from Hugo Health and ask them to stop your permission to have data transferred to this study. Please note, that if we have already shared information about you to other researchers, we will be unable to withdraw that data.

**Who should I contact if I have any questions?** If you have any questions about the study, or if you wish to withdraw your data from the study, please contact Dr. Kelli O’Laughlin at 206-543-3240

INSPIREREGISTRY@UW.EDU.

Subject’s statement

This study has been explained to me. I volunteer to take part in this research. I have had a chance to ask questions. If I have questions later about the research, or if I have been harmed by participating in this study, I can contact one of the researchers listed on the first page of this consent form. If I have questions about my rights as a research subject, I can call the Human Subjects Division at (206) 5430098 or call collect at (206) 221-5940. I give permission to the researchers to use my medical records as described in this consent form. -I will receive a copy of this consent form.

Click the following boxes to provide your consent for this research. You may participate in the COVID INSPIRE Registry and choose not to be contacted for future research. Contact for future research is optional. If you do not agree to participation, do not click a box. 1. Consent to participate in the COVID INSPIRE Registry:

□ I agree to volunteer for the COVID INSPIRE Registry

1. Consent for future research:

□ I agree to be contacted for future research

Typed name of participant                                                    Date
