# Supplementary material for: Study protocol for the Innovative Support for Patients with SARS-COV-2 Infections Registry (INSPIRE): A longitudinal study of the medium and long-term sequelae of SARS-CoV-2 infection
Source: PLoS One. 2022 Mar 3;17(3):e0264260. doi: 10.1371/journal.pone.0264260 (PMC8893622; doi:10.1371/journal.pone.0264260)
Supplement: S1 Appendix — (DOCX) [file pone.0264260.s002.docx]

INSPIRE Survey Specifications Version 8.0 (14 December 2021)

**APPENDIX A: INSPIRE Investigators**

**Rush University, Administrative Core & Enrolling Site**

**Study-wide Co- Principal Investigators and Site Core Investigators:** Bala Hota, MD; Robert A. Weinstein, MD

**Core research team:** Katherine Koo, MS

**Site Investigators:** Michael Gottlieb, MD, Site Principal Investigator

**Site research team:** Michelle Santangelo, MPH

**Yale University, Analytic Core & Enrolling Site**

**Core Investigators:** Arjun Venkatesh, MD, MBA, MHS; Erica Spatz MD, MHS; Andrew Ulrich, MD

**Core research team:** Zhenqiu Lin, PhD; Shu-Xia Li, PhD; Huihui Yu, PhD; Mengni Liu, MS; Jeremiah Kinsman, MPH

**Site Investigators:** Arjun Venkatesh, MD, MBA, MHS; Erica Spatz MD, MPH; Andrew Ulrich, MD

**Site research team:** Jeremiah Kinsman, MPH; Michelle Opare, BS

**University of Washington, Clinical Core & Enrolling Site**

**Core Investigators:** Graham Nichol, MD, Principal Investigator; Matthew Thompson, MD, MPH, DPhil, Principal Investigator

| **Core research team:** Jill Anderson, BSN, RN, Clinical Core Program Manager; Kari Black, BA, Grant & Finance Manager; Dana Morse BSN, Research Coordinator; Anoushka Fernandes BSc, Research Assistant.  **Site Investigators:** Kelli N. O’Laughlin, MD, MPH, Site Principal Investigator; Nikki Gentile, MD, PhD, Co-Investigator; Kari Stephens, PhD, Co-Investigator  **Site research team:** Rachel E. Geyer, MPH, Research Coordinator; Victoria Lyon, MPH, Program Manager; Sophie C. Morse, BA, BS, Research Assistant; Karen Adams, BA, Regulatory Specialist; Michael Willis, AS, BSHS, Research Assistant |
| --- |

**Thomas Jefferson University, Enrolling Site**

**Site Investigators:** Anna Marie Chang, MD, MSCE, Benjamin Slovis, MD, MA

**Site research team:** Morgan Kelly, BS, Alaina Hunt, BA, Kyle Norton, BA, Mubazar Ishfaq, BA, Paavali Hannikainen, BS, Melanie Chalfin, BA, Lindsey Shughart, Hailey Shughart, BA, Nicole Renzi, RN

**University of Texas Health Science Center at Houston, Enrolling Site**

**Site Investigators:** Mandy Hill, DrPH, MPH, Site Co-Principal Investigator; Ryan Huebinger Site, MD, Co-Principal Investigator; Summer Chavez, DO, MPH, MPM, Site Co-Investigator

**Site research team:** Elizabeth Vidales MD, MPH; Leslie Johnson BS

**University of Texas Southwestern Medical Center, Enrolling Site**

**Site Investigators:** Ahamed H. Idris, MD, PI; Samuel McDonald, MD, Co-I

**Site research team:** Paula Arellano-Cruz, Research Coordinator; David Gallegos, Research Associate

**University of California, Los Angeles, Enrolling Site**

**Site Investigators:** Joann Elmore, MD, MPH (site PI), Lauren Wisk, PhD

**Site research team:** Raul Moreno, BA, Dayna Clayton, BA, Annie Lee, PhD, Michelle L’Hommedieu, PhD, Chris Chandler, BA

**University of California, San Francisco, Enrolling Site**

**Site Investigators:** Robert Rodriguez, MD; Ralph C. Wang, MD, MAS; Juan Carlos Montoy, MD, PhD

**Site research team:** Robin Kemball, MPH; Virginia Chan, Cecilia Lara Chavez, Angela Wong

**Centers for Disease Control and Prevention (CDC)**

**Investigators:** Ian D. Plumb, MBBS, MSc; Aron J. Hall, DVM, MSPH; Sharon Saydah,

PhD
